# Infrared Small Target Detection Method Fusing Accurate Registration and Weighted Difference

**DOI:** 10.3390/s26082406

**Published:** 2026-04-14

**Authors:** Quan Liang, Teng Wang, Kefang Wang, Lixing Zhao, Xiaoyan Li, Fansheng Chen

**Affiliations:** 1 Hangzhou Institute for Advanced Study, Hangzhou 310024, China; 2 University of Chinese Academy of Sciences, Beijing 100049, China; 3 Key Laboratory of Intelligent Infrared Perception, Shanghai Institute of Technical Physics, Chinese Academy of Sciences, Shanghai 200083, China; 4 Shanghai Frontier Base of Intelligent Optoelectronics and Perception, Institute of Optoelectronics, Fudan University, Shanghai 200438, China

**Keywords:** small target detection, space-based thermal infrared remote sensing, sub-pixel registration, bidirectional whisk-broom imaging

## Abstract

Low-orbit thermal infrared bidirectional whisk-broom imaging offers wide-swath coverage and high spatial resolution for monitoring moving targets such as aircraft, but large scan angles and terrain undulation cause non-rigid geometric distortion and radiometric inconsistency between forward and backward scans. These effects generate strong clutter in difference images and degrade small and weak target detection. To address this problem, we propose an infrared small target detection method that fuses accurate registration and weighted difference. First, we propose a hybrid multi-scale registration algorithm that achieves coarse affine registration through sparse feature–point matching and then iteratively corrects nonlinear deformations by integrating a global grayscale-driven force with a local sparse-feature-guided force, yielding a registration error of 0.3281 pixels. On this basis, a multi-scale weighted convolutional morphological difference algorithm is proposed. A novel dual-structure hollow top-hat transform is constructed to accurately estimate the background, and a multi-directional convolution mechanism is introduced to effectively suppress anisotropic edge clutter and enhance target saliency. Experiments on SDGSAT-1 thermal infrared bidirectional whisk-broom data show an SCRG of 18.27, and a detection rate of 91.2% when the false alarm rate is below 0.15%. The method outperforms representative competing algorithms and provides a useful reference for space-based aerial moving target detection.

## 1. Introduction

With the rapid development of Earth observation technology, space-based infrared remote sensing has become a strategic capability for national security and airspace surveillance because of its all-time, all-weather, and passive sensing advantages. Airplanes are typical high-value moving targets, and their real-time in-orbit detection is of great importance for air traffic control, maritime search and rescue, and military reconnaissance [1,2]. However, limited by imaging distance and sensor resolution, aircraft in thermal infrared imagery usually appear as weak point-like targets with little geometric texture or shape information. They are therefore easily submerged in complex background clutter such as land–sea boundaries and bright urban heat sources, which makes detection extremely challenging [3,4].

Existing infrared small and weak target detection methods can be broadly divided into single-frame and multi-frame approaches [5,6]. Traditional single-frame methods rely on feature differences between target and background, including morphological filtering, local contrast measurement, and low-rank sparse decomposition [7,8,9]. To address the challenge of background estimation in complex scenes, Cui et al. further developed a hollow side window filtering (HSWF) algorithm. By integrating a heterogeneity-filtering saliency map with image-patch contrast measures, the method optimizes background estimation and effectively improves target extraction performance in complex cluttered backgrounds [10]. Although single-frame methods do not depend on temporal information, they often struggle to suppress strong structured clutter completely, which can cause false alarms. And some iterative optimization schemes remain computationally demanding for wide-swath remote sensing. By contrast, multi-frame methods exploit the spatiotemporal continuity of target motion and can in principle achieve better performance under low signal-to-clutter conditions [11]. For example, Pang et al. constructed a spatiotemporal fusion framework by combining spatial variance and temporal grayscale saliency to extract low-altitude, slow, and small targets under complex backgrounds [12]. Nevertheless, the effectiveness of multi-frame methods depends heavily on both registration accuracy and frame count. A lot of existing spatiotemporal algorithms require at least three consecutive frames to build stable background models or tensor constraints [13,14]. What’s more, conventional feature–point registration usually assumes rigid transformation and is therefore insufficient for correcting local nonlinear distortion for satellite platforms [15], which easily produces false target responses. Deep-learning methods have also achieved strong performance in recent years. For instance, RISTDnet combines handcrafted features with convolutional neural networks to improve robustness under low signal-to-noise conditions [16], but the demand for large annotated datasets conflicts with the scarcity of space-based infrared aircraft samples and limits generalization in real on-orbit applications.

The Sustainable Development Science Satellite-1 (SDGSAT-1), launched in 2021, provides a new data source for airplane detection. Its thermal infrared imager (TIS) offers a world-leading spatial resolution of 30 m [17,18,19] and adopts a distinctive bidirectional whisk-broom imaging mechanism that acquires a pair of forward and backward scanning images for the same region within a very short time interval. At the same time, this observation mode introduces more complex nonlinear geometric deformation and radiometric inconsistency caused by differences in viewing angle, which makes conventional detection methods less effective.

To address the above limitations, this study focuses on high-accuracy target detection for SDGSAT-1 bidirectional whisk-broom thermal infrared imagery. This study focuses on the problem of high-precision target detection using this emerging type of data source. It aims to overcome the limitations of conventional rigid registration models and to address the complex nonlinear deformations and radiometric discrepancies between forward- and backward-scan images. In addition, it seeks to develop a robust strategy for effectively suppressing dynamic edge clutter and accurately capturing weak moving targets under low signal-to-clutter conditions. By integrating high-precision physical alignment with refined differential detection, the proposed framework aims to exploit the value of bidirectional whisk-broom observations and support the practical application of next-generation wide-swath infrared remote sensing in complex dynamic scenes.

## 2. Methods

### 2.1. Overall Framework

**Figure 1:**
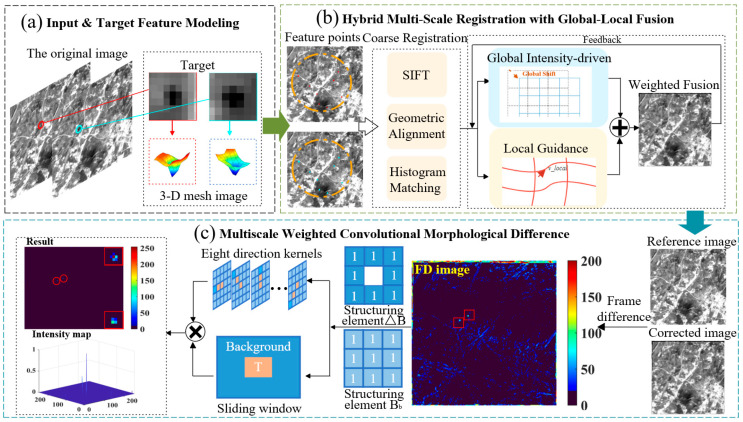
Framework of the infrared small target detection method fusing accurate registration and weighted difference.

To address the non-rigid geometric distortion and complex background clutter in forward and backward scanning images, we propose an infrared small target detection method fusing accurate registration and weighted difference. The overall framework is shown in Figure 1 and follows a cascaded strategy that performs high-precision alignment prior to refined detection and comprises two core functional modules.

The first module is a sub-pixel image registration stage that removes background misalignment caused by scan motion and sensor jitter. By combining geometric–radiometric coarse registration based on scale invariant feature transform (SIFT) features with iterative fine registration driven by a fused global–local dual flow field, the method corrects both global rigid displacement and local nonlinear deformation between forward and backward scanning images, thereby providing high-quality registered residual images for subsequent differencing.

The second module is a refined target detection stage that extracts low signal-to-clutter targets from the registered residual image. To address residual interpolation artifacts and background clutter after registration, a multiscale weighted convolutional morphological difference operator is constructed. This module integrates a novel dual-structure top hat transform, multi-directional convolutional suppression and local energy enhancement. It effectively suppresses anisotropic edge clutter while enhancing point–target signals, thereby enabling robust detection in complex dynamic backgrounds.

### 2.2. Hybrid Multi-Scale Registration with Global–Local Fusion (HMSR)

To improve the accuracy of aircraft detection on bidirectional whisk-broom imagery, a hybrid multi-scale registration algorithm is introduced to align the moving image to the reference image at sub-pixel precision. Conventional SIFT registration can compensate only for global rigid transformation and is insufficient for nonlinear deformation [20]. Therefore, a non-rigid deformation field is built on top of affine transformation, and high-accuracy linear and nonlinear alignment is achieved by combining the smoothness of a global gradient flow with the feature sensitivity of local sparse optical flow. The algorithm consists of two stages: SIFT-based coarse registration and global–local fusion iterative refinement. To provide a clear and intuitive explanation of the overall procedure, the detailed pseudocode of the proposed method is summarized in Algorithm 1.
**Algorithm 1** Hybrid Multi-Scale Registration with Global–Local Fusion**Input:** Reference image $I_{R}$, Image to be registered $I_{M}$. **Output:** Final registered image $I_{out}$, Cumulative deformation field u. 1: Extract sparse feature points $P={\left\{p_{i}\right\}}_{i=1}^{N}$ from $I_{R}$ and $I_{M}$ using SIFT. 2: Estimate affine transformation matrix $H_{aff}$ by minimizing Euclidean distance. 3: Obtain initially registered image $I_{M}^{\left(0\right)}=T(I_{M},H_{aff})$. 4: Perform radiometric correction via histogram specification on $I_{M}^{\left(0\right)}$ to get ${\tilde{I}}_{M}^{\left(0\right)}$ (Equation (2)). 5: **Initialize:** Iteration index k=0, cumulative deformation field $u^{\left(0\right)}=0$, learning rate $\alpha_{0}=0.6$, attenuation factor γ=0.95. 6: **While** not converged **do** 7: Calculate global gradient flow $v_{\mathrm{global}}$ using Demons-type energy (Equation (4)). 8: **For** each feature point $p_{i}\in P$ **do** 9:      Compute local micro-displacement $d_{i}$ via Lucas–Kanade optical flow (Equation (6)). 10: **End For** 11: Spread sparse displacements $\left\{d_{i}\right\}$ to dense local driving field $v_{\mathrm{lobal}}$ (Equation (7)). 12: Fuse global and local flows, and apply Gaussian smoothing $G_{\sigma_{k}}$ to obtain total displacement update $\Delta u^{\left(k\right)}$ (Equation (8)). 13: Update the cumulative deformation field: $u^{\left(k+1\right)}=u^{\left(k\right)}+\alpha_{k}\cdot \Delta u^{\left(k\right)}$. 14: Update the learning rate: $\alpha_{k+1}=\gamma \cdot \alpha_{k}$. 15: Warp image ${\tilde{I}}_{M}^{\left(k\right)}$ using $u^{\left(k+1\right)}$ 16: k=k+1. 17: **End While**

#### 2.2.1. SIFT-Based Coarse Registration

The purpose of the coarse registration stage is to remove large geometric displacement between the two images and to provide a good initialization for fine registration. Sparse feature points $P={\left\{p_{i}\right\}}_{i=1}^{N}$ are first extracted and matched by the SIFT operator, and an affine transformation matrix is estimated by minimizing the Euclidean distance between matched feature pairs:

(1) $$H_{aff}=\arg\underset{H}{\min}\sum_{i=1}^{N}||z_{R}^{i}-H\cdot z_{M}^{i}{∥}^{2}$$

where $H_{aff}$ denotes the 3 × 3 affine transformation matrix to be estimated, $z_{R}^{i}$ and $z_{M}^{i}$ denote the homogeneous coordinate vectors of the *i*th matched point pair in the reference image and image to be registered, and $||\cdot {∥}^{2}$ denotes the L2 norm.

The image to be registered is then spatially transformed by $H_{aff}$ to obtain an initial registered image $I_{M}^{\left(0\right)}=T\left(I_{M},H_{aff}\right)$, where T denotes spatial transformation operator.

Because the two images are acquired at different moments of the bidirectional scan, nonlinear grayscale differences often remain. To satisfy the grayscale constancy assumption required by the subsequent variational optical flow refinement, histogram specification is used to perform radiometric correction on the coarsely registered image. Specifically, grayscale-level probability density functions $p_{r}(k)$ and $p_{m}(k)$ are constructed for the reference image $I_{R}$ and the geometrically registered image $I_{M}^{\left(0\right)}$, respectively. Using the gray-level mapping function Μ(g). The corrected image is finally obtained as:

(2) $${\tilde{I}}_{M}^{\left(0\right)}(x,y)=Μ(I_{M}^{\left(0\right)}(x,y))=C_{R}^{-1}(C_{M}(I_{M}^{\left(0\right)}(x,y)))$$

where $C_{M(g)}=\sum_{k=0}^{g}p_{m}(k)$ denotes the cumulative distribution function of the image to be registered, $C_{R(g)}=\sum_{k=0}^{g}p_{r}(k)$ denotes the cumulative distribution function of the reference image, and $C_{R}^{-1}$ denotes its inverse transform.

#### 2.2.2. Global–Local Fusion Iterative Optimization

In the fine registration stage, a dense deformation field u(x,y) is constructed to eliminate the remaining nonlinear distortion. The deformation field is estimated iteratively. At the *k*th iteration, the total displacement $\Delta u^{\left(k\right)}$ update is obtained by fusing a global driving force and a local feature-guidance force. The update logic is as follows:

After radiometric correction, the image pair approximately satisfies the grayscale constancy assumption. A Demons-type formulation is then used to compute the global driving force [21]. The pixelwise grayscale squared-difference energy is defined as follows:

(3) $$E_{\mathrm{global}}=\frac{1}{2}{\left(I_{M}^{\left(k\right)}(x,y)-I_{R}(x,y)\right)}^{2}$$

where ${\tilde{I}}_{M}^{\left(k\right)}(x,y)$ denotes the grayscale value of the image to be registered at the *k*th iteration, and $I_{R}(x,y)$ denotes the grayscale value of the reference image.

According to a Gauss–Newton optimization strategy, the global displacement increment that minimizes the energy can be expressed as:

(4) $$v_{\mathrm{global}}(x,y)=-\frac{\left(I_{M}^{\left(k\right)}(x,y)-I_{R}(x,y)\right)\cdot \nabla I_{R}(x,y)}{∥\nabla I_{R}(x,y){∥}^{2}+\epsilon }$$

where $\nabla I_{R}(x,y)$ denotes the gradient of the reference image, and ϵ is a small positive constant for numerical stability.

The global gradient flow drives smooth large-range deformation. To capture high-frequency details around feature points, a Lucas–Kanade optical flow equation is solved in the neighborhood of each SIFT feature point $p_{i}$ [22]. For the *i*th feature point, the local optimal micro-displacement $d_{i}$ is obtained by minimizing the following optical-flow constraint within a local window $\Omega_{i}$.

(5) $$\underset{d_{i}}{\min}\sum_{x,y\in \Omega_{i}}{\left[\nabla I_{R}(x,y)\cdot d_{i}+\left(I_{M}^{\left(k\right)}(x,y)-I_{R}(x,y)\right)\right]}^{2}$$

The closed-form least-squares solution is:

(6) $$d_{i}={\left(\sum_{x,y\in \Omega_{i}}\nabla I_{R}\nabla I_{R}^{T}\right)}^{-1}\left(\sum_{x,y\in \Omega_{i}}-\nabla I_{R}\left({\tilde{I}}_{M}^{\left(k\right)}-I_{R}\right)\right)$$

After the sparse displacement set is obtained, it is spread to the entire image through a nearest-neighbor interpolation operator Φ to form the local driving field:

(7) $$v_{\mathrm{local}}(x)=\Phi \left({\left\{p_{i},d_{i}\right\}}_{i=1}^{N}\right)$$

To balance global consistency and local accuracy, the global and local fields are linearly fused and then regularized by Gaussian smoothing to guarantee physical continuity. The final displacement update at iteration k is defined as:

(8) $$\Delta u^{\left(k\right)}=G_{\sigma_{k}}*\left(\lambda_{g}v_{\mathrm{global}}+\lambda_{l}v_{\mathrm{local}}\right)$$

where $\lambda_{g},\lambda_{l}$ are the fusion weights of the global and local flows, satisfying the constraint $\lambda_{g}+\lambda_{l}=1$. $G_{\sigma_{k}}$ is a Gaussian smoothing kernel, ∗ denotes the convolution operation and the standard deviation $\sigma_{k}$ with the iteration number.

The cumulative deformation field is then updated as follows:

(9) $$u^{\left(k+1\right)}=u^{\left(k\right)}+\alpha_{k}\cdot \Delta u^{\left(k\right)}$$

(10) $$\alpha_{k+1}=\gamma \cdot \alpha_{k}$$

where $u^{\left(k\right)}$ is the accumulated deformation field at the *k*th iteration, $\alpha_{k}$ denotes the learning rate, and γ is the step-size attenuation factor. In this study, the initial learning rate is set to 0.6 and the attenuation factor is set to 0.95.

### 2.3. Multiscale Weighted Convolutional Morphological Difference (MWCMD)

Although HMSR achieves sub-pixel registration and corrects global geometric misalignment, radiometric noise and high-frequency background clutter may still remain because of focal-plane nonuniformity and differences in observation angle. To capture weak targets under low signal-to-clutter conditions, a refined detection algorithm is further developed.

#### 2.3.1. Image Enhancement Preprocessing

Small and weak infrared targets are often submerged in strong background radiation and have very limited energy. Therefore, contrast enhancement is performed first. A percentage-clipping linear stretch strategy is adopted by truncating a small portion of pixels at both ends of the histogram and mapping the major grayscale interval to the target dynamic range. The enhanced image $I_{str}$ is obtained:

(11) $$I_{str}=\frac{gray-P_{down}}{P_{up}-P_{down}}\times (max_{out}-min_{out})+min_{out}$$

where gray denotes the original grayscale value, $P_{down}$ and $P_{up}$ denote the low and high clipping percentiles determined from the histogram, and $min_{out}$ and $max_{out}$ denote the minimum and maximum grayscale levels of the output image.

Using the enhanced forward and backward scanning images, an initial difference image is computed for subsequent background suppression:

(12) $$I_{diff}(x,y)=\left|I_{r\_str}(x,y)-I_{i\_str}(x,y)\right|$$

where $I_{r\_str}$ and $I_{i\_str}$ denote the enhanced and registered forward scanning images and backward scanning images, respectively.

#### 2.3.2. Fine Target Detection Using MWCMD

To suppress strong edge clutter remaining in the difference image, an MWCMD operator is constructed. The operator integrates a new morphological filter, multi-directional convolution suppression, and local contrast enhancement so that point-like targets are strengthened while anisotropic edge structures are removed.

**Figure 2:**
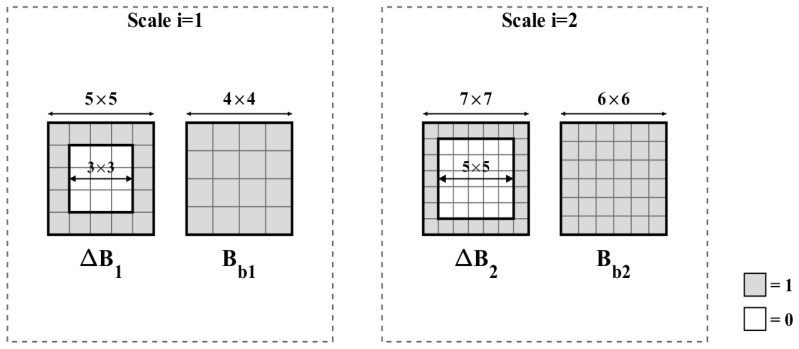
Multi-scale structuring element pairs.

Conventional structuring elements often fail to achieve a satisfactory balance between background suppression and target preservation. Considering that weak infrared small targets in the present imagery typically exhibit compact responses with effective spatial extents of approximately 3 × 3 and 5 × 5 pixels, and that their energy is concentrated near the center and decays toward the boundary, two multi-scale dual-structure element pairs are designed on the basis of the NWTH [23,24], as shown in Figure 2.

At each scale, the corresponding structuring element pair is configured to match the target core while sampling the surrounding background within a protected neighborhood, thereby improving background estimation without substantially attenuating the target response. The saliency feature map at the *i*th scale is then defined as:

(13) $$M_{i}(x,y)=I_{diff}(x,y)-\left[(I_{diff}(x,y)⊕\Delta B_{i})⊖B_{bi}\right]$$

where $M_{i}(x,y)$ denotes the saliency feature map at the *i*th scale, and the morphological dilation and erosion operators are represented by ⊕ and ⊖, respectively.

Although the above filter can suppress smooth background, grayscale discontinuities introduced by resampling near registration boundaries may still produce anisotropic line-like edges. To remove such interference, eight directional derivative convolution kernels are constructed by exploiting the isotropic nature of point targets. The minimum response among all directional convolutions is taken to define the multi-directional convolution suppression index (MCI):

(14) $$MCI_{i}(x,y)=\underset{j=1,\ldots ,8}{\min}((M_{i}*k_{j})(x,y)\times M_{i}(x,y))$$

where $k_{j}$ denotes the *j*th directional kernel and ∗ denotes convolution.

**Figure 3:**
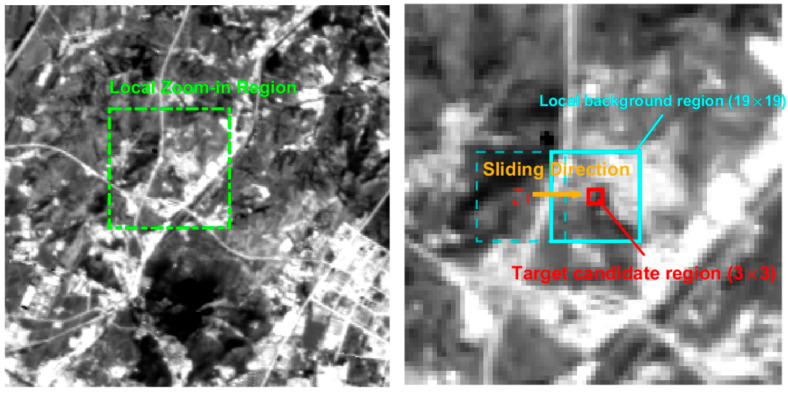
Schematic diagram of the global sliding window strategy.

To further improve the signal-to-clutter ratio, a nested-window model is employed to compute local energy differences using the global sliding-window strategy illustrated in Figure 3. Specifically, the sliding window consists of an inner target window $W_{\mathrm{tar}}$ and an outer annular background window $W_{\mathrm{bkg}}$. In this study, $W_{\mathrm{tar}}$ is set to 3 × 3, while $W_{\mathrm{bkg}}$ is defined within a 19 × 19 local neighborhood. The nested-window structure is slid pixel by pixel over the morphological feature map $M_{i}$. At each location (x, y), the local energy difference is computed from the mean intensity difference between the target window and its surrounding background as follows:

(15) $$D(x,y)=\left\{\begin{array}{ccc} {\left|\mu_{\mathrm{tar}}(x,y)-\mu_{b}(x,y)\right|}^{2} & , & \mu_{\mathrm{tar}}>\mu_{\mathrm{bkg}}\\ 0 & , & \mu_{\mathrm{tar}}\leq \mu_{\mathrm{bkg}} \end{array}\right.$$

(16) $$MDI_{i}(x,y)=D(M_{i}(x,y))$$

where $\mu_{\mathrm{tar}}$ and $\mu_{\mathrm{bkg}}$ represent the mean grayscale values of the morphological feature map $M_{i}$ inside the target window $W_{\mathrm{tar}}$ and the background window $W_{\mathrm{bkg}}$, and MDI denotes the local contrast response.

Finally, the MCI feature that emphasizes morphological anisotropy suppression and the MDI feature that emphasizes local energy contrast are fused. The maximum response across scales is taken to adapt to target-size variation, yielding the final response map:

(17) $$MWCMD(x,y)=\underset{i}{\max}\{MDI_{i}(x,y)\times MCI_{i}(x,y)\}$$

The experiments use two scales to cover common target sizes of 3 × 3 and 5 × 5 pixels, following the structural-element design of the original NWTH method. An adaptive threshold is then used to segment the response map:

(18) $$Th=\mu_{map}+k\sigma_{map}$$

where $\mu_{map}$ and $\sigma_{map}$ are the mean and standard deviation of the final response map, respectively, and k is an empirical coefficient controlling the false alarm rate. In this study, k is set to 40. The thresholded binary image is taken as the final detection result.

### 2.4. Dataset

**Figure 4:**
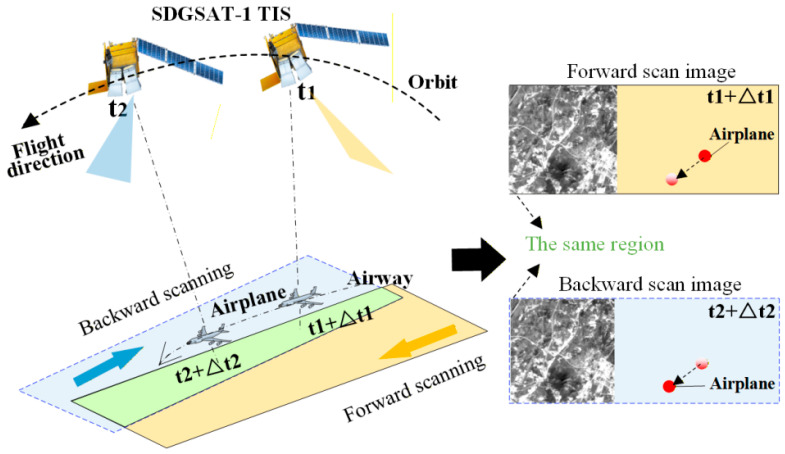
Schematic diagram of target observation via SDGSAT-1 TIS bi-directional whisk-broom imaging.

To validate the effectiveness and robustness of the proposed method, a real-scene airplane dataset consisting of forward and backward scanning image pairs was built from the SDGSAT-1 TIS observations. As shown in Figure 4, the high-frequency whisk-broom scanning mirror enables quasi-simultaneous bidirectional observations of the same ground target within a very short time interval. This observation mode not only improves data acquisition efficiency but also preserves the prominent non-rigid geometric deformation and radiometric inconsistency caused by different viewing angles, making it well suited for testing algorithms under complex dynamic conditions.

**Figure 5:**
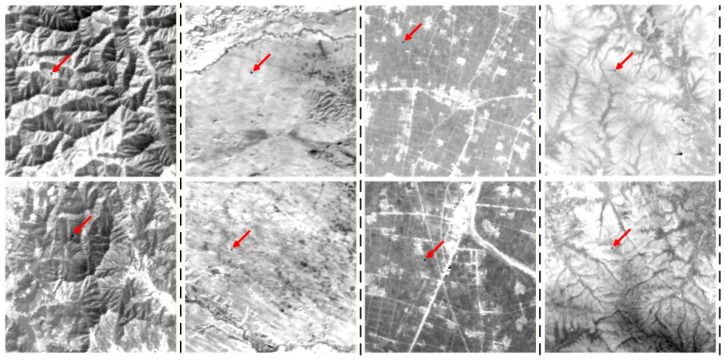
Images of complex land background scenes. The red arrows indicate the locations of targets in complex backgrounds.

To build a representative and challenging benchmark, a strict sample selection strategy was adopted. Because the radiation background over the sea is relatively uniform and does not sufficiently test clutter suppression under extreme interference, those simpler samples were excluded. Instead, a core validation dataset containing 20 groups of high-complexity land-background images was assembled. As illustrated in Figure 5, the dataset covers multiple typical scenarios with strong surface heterogeneity and pronounced edge clutter, from bright urban thermal sources to complicated mountainous textures. These images provide a comprehensive basis for evaluating the proposed detector under low signal-to-clutter conditions and strong background interference.

### 2.5. Evaluation Metrics

In the experiments, the forward-scan image is used as the reference image, and the backward scanning image is treated as the image to be registered. Registration accuracy is evaluated by the root mean square error (RMSE) of matched feature–point pairs:

(19) $$RMSE=\sqrt{\frac{1}{N}\sum_{i=1}^{N}{\left(x_{i}-x_{i}^{\prime }\right)}^{2}+{\left(y_{i}-y_{i}^{\prime }\right)}^{2}}$$

where $\left(x_{i},y_{i}\right)$ and $\left(x_{i}^{\prime },y_{i}^{\prime }\right)$ denote the coordinates of the *i*th feature point in the reference image and the registered image, respectively. N is the number of feature–point pairs.

To evaluate the detection performance, the signal-to-clutter ratio gain (SCRG) is used to quantify target enhancement, and the background suppression factor (BSF) is used to measure clutter suppression. The corresponding definitions are:

(20) $$BSF=\frac{\sigma_{in}}{\sigma_{out}+c},SCR=\left|\frac{\mu_{tar}-\mu_{bk}}{\sigma_{bk}+c}\right|$$

(21) $$SCRG=\frac{SCR_{out}}{SCR_{in}}$$

where $SCR_{in}$ and $SCR_{out}$ denote the signal-to-clutter ratios before and after processing, $\sigma_{in}$ and $\sigma_{out}$ denote the corresponding background standard deviations, c is a small positive constant preventing division by zero, $\mu_{tar}$ denotes the mean grayscale of the target region, and $\mu_{bk}$ and $\sigma_{bk}$ denote the mean and standard deviation of the background region, respectively.

To further evaluate detection accuracy, the true positive rate (TPR) and false positive rate (FPR) are defined as:

(22) $$TPR=\frac{N_{d}}{N_{tar}},FPR=\frac{N_{f}}{N_{t}}$$

where $N_{d}$ is the number of correctly detected targets, $N_{tar}$ is the total number of true targets in the current image, $N_{f}$ is the number of false detections, and $N_{t}$ is the total number of detected pixels.

## 3. Results

The methods are implemented using MATLAB R2023a on a CPU (Intel Core i7-13620H) with 16 GB of RAM. A stepwise validation strategy was used to comprehensively evaluate the proposed method on forward and backward scanning image pairs. First, the accuracy of the HMSR registration algorithm was quantitatively verified. To further validate the effectiveness of the HMSR registration module, the proposed MWCMD detector was combined with a mainstream registration algorithm, GLF-Reg, forming the GLF-MWCMD baseline. Then, to compare different back-end detection algorithms fairly on a common high-precision registration basis, HMSR was cascaded with four representative detectors: frame differencing (FD) [23], new white top-hat (NWTH) [24], partial sum of tensor nuclear norm (PSTNN) [25], and tri-layer local contrast measure (TLLCM) [26]. The corresponding combinations are referred to as HMSR-FD, HMSR-NWTH, HMSR-PSTNN, and HMSR-TLLCM. Qualitative visualization, quantitative metrics, and receiver operating characteristic (ROC) curves were then used for comparison against FD and the registered baselines.

### 3.1. Registration Performance

#### 3.1.1. Parameter Sensitivity Analysis

Before evaluating the final registration accuracy, a parameter sensitivity analysis was conducted to determine the optimal fusion weights $\lambda_{g}$ and $\lambda_{l}$, and to verify the robustness of the HMSR algorithm across diverse complex backgrounds. To maintain the physical stability of the displacement update and avoid unwanted scaling of the deformation field, the weights were naturally constrained by $\lambda_{g}+\lambda_{l}=1$. We evaluated the final registration RMSE across six representative validation scenes by varying $\lambda_{g}$ from 0 to 1 at intervals of 0.1.

**Figure 6:**
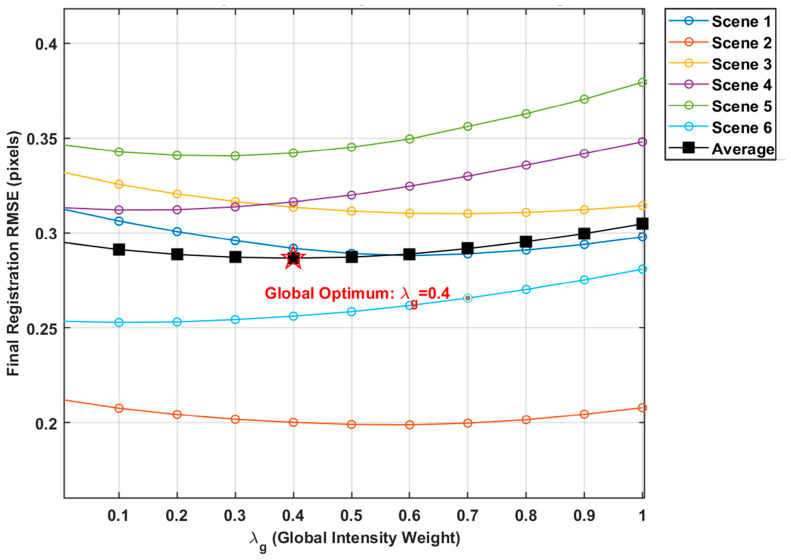
Parameter sensitivity and robustness analysis of the global intensity weight.

As illustrated in Figure 6, the sensitivity of the registration performance was first evaluated across six typical validation scenes with varying degrees of background complexity. Although the initial geometric distortions and absolute registration errors vary depending on the inherent complexity of each scene, the error curves exhibit a highly consistent, bowl-shaped trend. The average RMSE curve clearly demonstrates that the registration error reaches its global optimum when $\lambda_{g}=0.4$. This indicates that allocating a slightly higher weight to local feature guidance, while maintaining global physical continuity, provides the optimal sub-pixel alignment for bidirectional whisk-broom imagery. Furthermore, the RMSE remains stable and near-optimal within the range of $\lambda_{g}\in [0.3,0.5]$, proving that the proposed fixed-parameter setting is highly robust to scene variations.

#### 3.1.2. Accuracy Evaluation

**Figure 7:**
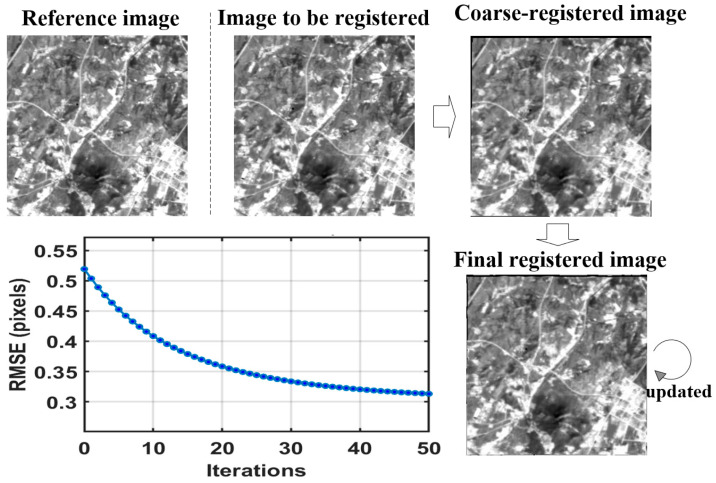
Results of the HMSR algorithm.

High-precision background alignment is a prerequisite for subsequent difference-based detection. Figure 7 and Table 1 jointly illustrate the convergence behavior and registration accuracy of the proposed HMSR method. As shown by the feature–point RMSE curve in Figure 6, the residual registration error decreases steadily with the iterative update of the global–local dual-flow field and converges after approximately 40 iterations, without noticeable oscillation or divergence. To further validate the effectiveness of the proposed method, a recently developed coarse-to-fine registration method that integrates global templates with local features (denoted as GLF-Reg) [27] is introduced for comparison.

Table 1 shows that the original image pair has a large displacement of 2.9232 pixels, indicating significant geometric misalignment. After SIFT-based coarse registration, the accuracy improves by about 85.2% relative to the unregistered state, which effectively removes the global rigid displacement. On this basis, the proposed non-rigid refinement further improves the registration accuracy by about 23.9%, finally achieving an RMSE of 0.3281 pixels. For comparison, the recently introduced GLF-Reg method attains an RMSE of 0.3967 pixels, which is higher than that of the proposed method.

These results indicate that the proposed method is more effective in correcting nonlinear sub-pixel distortion and local warping, thereby providing a more reliable basis for subsequent target detection.

### 3.2. Parameter Analysis for Detection Module

#### 3.2.1. Sensitivity of Structuring Element (SE) Sizes

In the design of the MWCMD operator, the size of the SE directly determines the accuracy of background estimation and the preservation of target energy. To identify the optimal parameter configuration, an ablation study was conducted on four groups of structuring element combinations with varying scales, designated SE1 through SE4. To ensure the consistency and fairness of the parameter evaluation, this ablation study was conducted on the same six representative complex scenes used in the registration parameter analysis. Given that targets in low-orbit thermal infrared whisk-broom imagery typically exhibit spatial extents of approximately 3 × 3 to 5 × 5 pixels, the inner hollow regions for all test pairs were fixed at 3 × 3 and 5 × 5 to maximize the preservation of the weak target’s core energy.

The specific parameter configurations are as follows: the SE1 combination consists of 5 × 5/4 × 4 and 7 × 7/6 × 6 structuring element pairs; SE2 utilizes 7 × 7/4 × 4 and 9 × 9/6 × 6 pairs; SE3 employs 9 × 9/6 × 6 and 11 × 11/8 × 8 pairs; and SE4 uses the largest configuration of 11 × 11/8 × 8 and 13 × 13/11 × 11 pairs.

**Figure 8:**
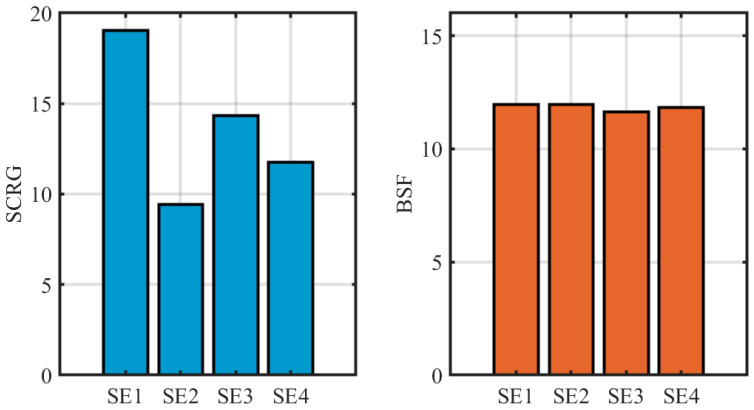
Impact of different structuring element sizes on SCRG and BSF performance.

The experimental evaluation results are illustrated in Figure 8. Regarding the SCRG metric, the SE1 configuration demonstrates a marked superiority, providing a significantly higher target enhancement effect compared to the other groups. As the SE size increases from SE1 to SE4, the SCRG shows a clear declining trend. This phenomenon indicates that excessively large background sampling windows are prone to introducing heterogeneous clutter interference from regions distant from the target, thereby diluting the precision of the local contrast estimation. Conversely, the BSF remains highly stable across the four experimental groups with only marginal fluctuations. This suggests that changes in structuring element size have a relatively limited impact on the global suppression of the background standard deviation.

In conclusion, the SE1 configuration (5 × 5/4 × 4 and 7 × 7/6 × 6) provides the strongest target enhancement response while maintaining a robust background suppression capability. Consequently, SE1 was selected as the standard parameter configuration for the MWCMD operator in all subsequent performance comparison experiments.

#### 3.2.2. Trade-Off Analysis of Empirical Coefficient k

**Figure 9:**
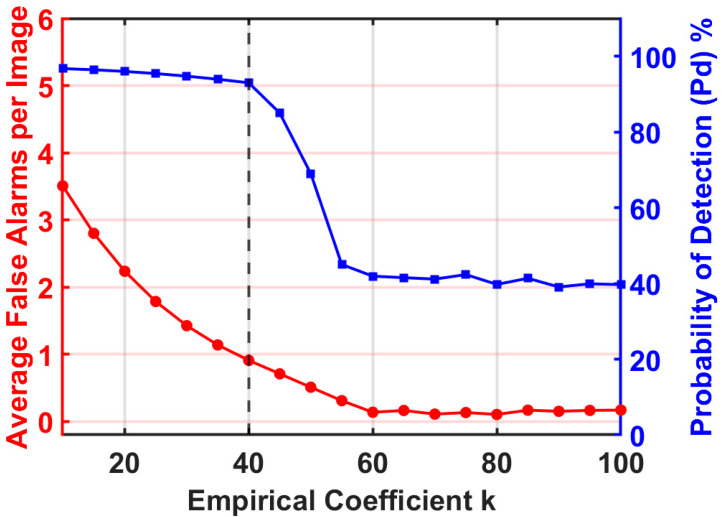
Trade-off analysis for varying empirical coefficient k.

The selection of the empirical coefficient k in Equation (18) is a critical factor that determines the segmentation threshold and directly influences the balance between detection sensitivity and false alarm suppression. To identify the optimal value, a comprehensive sensitivity analysis was conducted by varying k from 10 to 100, as illustrated in Figure 9. The dual-metric curves reveal a clear trade-off: when k < 30, the lenient threshold fails to suppress residual background clutter, resulting in a high average false alarm rate. Conversely, while the Pd remains at a high plateau exceeding 90% for k = 40, it suffers a sharp decline once k exceeds 45, as the overly aggressive threshold begins to eliminate the energy of targets.

The intersection of these trends identifies k = 40 as the optimal “elbow point”. At this setting, the average false alarms are significantly suppressed to approximately one per image while the detection probability is still maintained at its maximum level. This balance ensures high detection purity without compromising the algorithm’s sensitivity to target signals under low signal-to-clutter conditions. Consequently, k = 40 is selected as the standard coefficient for the proposed method to achieve robust performance across diverse complex scenes.

### 3.3. Ablation Study and Synergistic Analysis

To thoroughly evaluate the individual contributions of the core sub-modules within the proposed HMSR and MWCMD algorithms, a comprehensive ablation study was conducted across all representative complex scenes. To accurately isolate the performance gain of each component, a strict control–variable strategy was employed: when evaluating the registration module, the downstream detection algorithm was fixed to the complete MWCMD operator; conversely, when evaluating the detection module, the input was fixed to the perfectly registered difference images generated by the complete HMSR.

#### 3.3.1. Registration Module Ablation

Table 2 presents the quantitative results of the progressive registration stages. Without any registration, the severe geometric misalignment between the forward and backward scans generates a massive amount of dynamic differential clutter. Under this condition, even the powerful MWCMD detector suffers a significant performance drop, achieving a TPR of only 61.4% and an SCRG of 5.5134. By sequentially introducing SIFT coarse registration and global optical flow, the global rigid displacement is removed, and the RMSE decreases to 0.3494 pixels. Finally, the proposed Full HMSR, which ingeniously integrates global and local optical flows, achieves the highest sub-pixel alignment accuracy. This high-precision physical alignment essentially cleans the dynamic difference map from its root, providing an optimal foundation for the downstream detector to achieve the highest SCRG of 18.2704 and elevate the TPR to 91.2% while maintaining an extremely low false positive rate.

#### 3.3.2. Detection Module Ablation

Table 3 details the performance metrics for the detection module ablation. In this group of experiments, the input is strictly fixed to the optimally registered difference images. The results indicate that the basic NWTH transform can capture most targets with a TPR of 90.3% by adopting a low threshold. However, it struggles to suppress the complex anisotropic edge artifacts remaining after registration, which leads to a poor BSF of 5.5132. The subsequent introduction of multi-directional convolution suppression effectively filters out these line-like false alarms. This integrated NWTH and MCI approach significantly boosts the SCRG to 13.7925. By further incorporating local energy enhancement, the proposed full MWCMD operator maximizes the point–target saliency. Consequently, it achieves the best overall performance, dramatically improving the SCRG to 18.2704 and the BSF to 12.9367, while simultaneously pulling the TPR up to 91.2% and minimizing the FPR to 0.15%.

These ablation results strongly verify that the performance leap of the proposed framework relies on the synergistic advantages of both core modules: HMSR provides the essential geometric correction to remove pseudo-edges, while MWCMD leverages morphological and energy-contrast filtering to extract weak targets from the residual noise.

### 3.4. Comparison with Competing Algorithms

**Figure 10:**
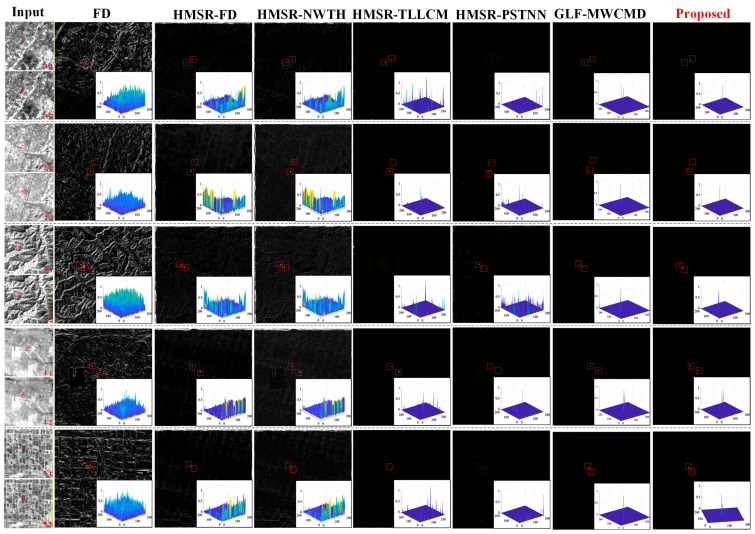
Visual comparison of detection results under typical scenes using different algorithms.

Figure 10 presents two-dimensional saliency maps and three-dimensional grayscale distributions for five representative complex scenes. For clarity, red solid boxes denote true targets successfully captured by each algorithm, whereas red dashed boxes indicate missed targets. The FD method suffers severely from background motion because it does not include effective registration, and its output is dominated by bright pseudo responses produced by background artifacts. Furthermore, due to its relatively lower registration accuracy, the GLF-MWCMD combination still suffers from noticeable false alarms caused by residual pseudo-edges, which is particularly evident in the first visualized scene.

Although HMSR-FD and HMSR-NWTH suppress large-scale clutter to some extent after registration, significant interpolation-edge artifacts or insufficient suppression of strong edges still remain. The more advanced HMSR-PSTNN and HMSR-TLLCM perform well in relatively flat regions, but they still generate false high-frequency responses in complex textured backgrounds. By contrast, the proposed method achieves the most thorough background suppression, owing to the strong capability of the MWCMD operator to suppress anisotropic edges. As shown in the three-dimensional mesh plots, the background region is almost completely smoothed to zero gray level, whereas the target region remains as a sharp impulse-like response. These results demonstrate that the proposed method possesses superior capability in both suppressing background clutter and enhancing target saliency.

The same conclusion is further supported by the quantitative results in Table 4, which report SCRG, BSF, and execution time for all compared methods. The proposed method achieves the highest SCRG of 18.2704, demonstrating markedly stronger target enhancement capability. Although HMSR-PSTNN yields the highest BSF, this is mainly attributable to over-suppression caused by its low-rank-plus-sparse formulation under strong and complex clutter. Such an apparently “black background” may increase BSF numerically at the expense of target integrity and can therefore lead to missed detections. In contrast, the proposed method attains a high BSF of 12.9367 while preserving the highest SCRG, indicating a better balance between background suppression and true-target preservation. In terms of computational cost, baseline methods like MWCMD require very short execution times but provide severely limited precision. Similarly, when compared to GLF-MWCMD, although GLF-MWCMD benefits from a shorter execution time, its lower precision in target enhancement compromises target integrity, ultimately leading to slightly degraded final detection results. Because the proposed method is developed on the basis of HMSR-NWTH with additional enhancement modules, its execution time (1.4862 s) is slightly longer than that of HMSR-NWTH (1.3587 s). However, this marginal increase in runtime brings substantial performance gains. Moreover, the proposed method remains more efficient than HMSR-TLLCM (1.9851 s) and HMSR-PSTNN (2.3472 s), while delivering superior overall detection performance. These results demonstrate that the proposed method achieves a more favorable trade-off among target enhancement, clutter suppression, and computational efficiency.

To further analyze the computational cost and identify the bottleneck for future on-board real-time applications, the execution time of the proposed method is broken down into sub-modules. Since the baseline MWCMD algorithm consists exclusively of the detection module, its execution time of 0.2219 s serves as a precise reference for the time consumption of our detection phase. Deducting this from the total execution time of 1.4862 s, the image registration module requires approximately 1.2643 s. This breakdown reveals that the registration process constitutes the primary computational bottleneck, accounting for roughly 85.1% of the total time, while the detection module remains highly efficient. Clarifying this time distribution provides a solid foundation for future lightweight improvements aimed at real-time processing.

**Figure 11:**
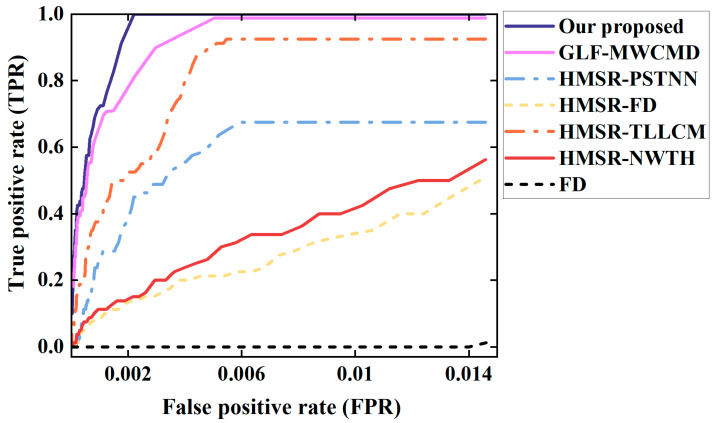
ROC curves showing the relationship between detection rate and false alarm rate for different algorithms.

The ROC curves in Figure 11 show that the proposed method consistently envelops the competing algorithms and stays closest to the upper-left corner of the coordinate system. In the low-false-alarm region that matters most in practice, its curve rises most rapidly, indicating that the highest detection rate can be achieved under the same false-alarm constraint. Therefore, from visual inspection, quantitative metrics, and ROC analysis, the proposed method demonstrates the best overall performance for handling complex geometric distortion and background interference in bidirectional whisk-broom images.

The experimental results indicate that accurate physical alignment and refined residual detection should be treated as a coupled problem rather than as independent stages for bidirectional whisk-broom infrared imagery. To explicitly distinguish the performance contributions of the registration and detection modules, we evaluated the GLF-MWCMD configuration, which pairs a mainstream registration algorithm with our proposed detector. As illustrated in the ROC curves, GLF-MWCMD demonstrates strong detection capabilities, confirming the intrinsic effectiveness of the MWCMD module in extracting targets. However, it exhibits a noticeable deficiency in controlling the false positive rate compared to our complete proposed method. When the registration error is reduced to the sub-pixel level by the HMSR module, a large fraction of the pseudo responses in the difference image disappears. This substantially improves the operating conditions for the downstream detector and explains why the proposed method can outperform classical local-contrast and tensor-based algorithms even though it uses only a pair of forward and backward scanning images. This confirms the synergistic advantages of the two core modules: HMSR provides an optimal, strictly aligned foundation to minimize dynamic clutter, while MWCMD ensures robust target extraction.

Another important observation is that strong background suppression alone does not guarantee the best detection result. In complex land scenes, high BSF values can be achieved by aggressively suppressing both clutter and target energy, which may increase the probability of missed detections. The proposed MWCMD operator addresses this trade-off by combining multi-scale morphology, multi-directional edge suppression, and local energy contrast. The resulting detector retains isotropic point–target responses while suppressing anisotropic edge structures, which is particularly useful for the dynamic edge clutter generated by whisk-broom scanning geometry.

## 4. Conclusions

This paper proposed an infrared small target detection method that fuses accurate registration and weighted difference to address non-rigid distortion and complex background interference in space-based thermal infrared bidirectional whisk-broom imaging. The core idea is to achieve sub-pixel physical alignment through a global–local fusion strategy together with radiometric correction, and then to exploit the multi-scale morphological difference between target and background through a weighted convolutional filtering framework for accurate target segmentation. Compared with representative small-target detectors, the proposed method exhibits superior target capture capability and false-alarm suppression over a variety of complex scenes. The study confirms that, in the absence of reliable prior texture information, suppressing differential artifacts through high-precision geometric correction is a key route to improving weak-target detection under low signal-to-clutter conditions.

Although the method achieves a substantial gain in accuracy, the iterative registration process increases computational cost. Future research will therefore focus on the trade-off between precision and efficiency for large-format remote sensing imagery and will explore lightweight and parallel acceleration schemes as well as fast registration strategies combined with deep-learning optical flow estimation for on-board real-time processing.

## Figures and Tables

**Table 1:** Quantitative evaluation of registration accuracy at different stages (RMSE/pixels).

Metric	Unregistered	Coarse Registration	Fine Registration (Ours)	GLF-Reg
RMSE	2.9232	0.4313	0.3281	0.3967

**Table 2:** Quantitative evaluation of different configurations within the proposed HMSR registration module.

Registration Stage	RMSE	SCRG	BSF	TPR (%)	FPR (%)
Unregistered	2.9232	5.5134	11.8923	61.4	0.61
SIFT Coarse	0.4313	6.7518	12.6815	89.7	0.39
SIFT & Global Optical Flow	0.3494	8.0052	12.9042	90.5	0.23
HMSR	0.3281	18.2704	12.9367	91.2	0.15

**Table 3:** Quantitative evaluation of different components within the proposed MWCMD detection module.

Detection Stage	SCRG	BSF	TPR (%)	FPR (%)
Basic NWTH	7.8741	5.5132	90.3	0.34
NWTH + MCI	13.7925	6.7385	91.2	0.28
MWCMD	18.2704	12.9367	91.2	0.15

**Table 4:** Quantitative comparison of SCRG, BSF and execution times among different detection algorithms. The bold numbers indicate the best performance in each column.

Method	SCRG	BSF	Execution Times (s)
FD	0.7164	2.3109	**0.01421**
MWCMD	5.1428	10.0854	0.2219
HMSR-FD	0.9295	5.1495	1.07234
HMSR-NWTH	1.1093	5.6728	1.3587
HMSR-PSTNN	2.6905	**13.5048**	2.3472
HMSR-TLLCM	5.9237	9.4283	1.9851
GLF-MWCMD	15.2315	10.1456	1.1809
Proposed	**18.2704**	12.9367	1.4862

## Data Availability

The source manuscript did not provide a public repository or accession number for the SDGSAT-1 TIS dataset. Data access details should be confirmed and completed by the authors before submission.

## Abbreviations

The following abbreviations are used in this manuscript:

SDGSAT-1	Sustainable Development Science Satellite-1
TIS	Thermal infrared imager of SDGSAT-1
SIFT	Scale-Invariant Feature Transform
HMSR	Hybrid Multi-Scale Registration with Global–Local Fusion
MWCMD	Multiscale Weighted Convolutional Morphological Difference
MCI	Multi-Directional Convolution Suppression Index
FD	Frame Differencing
NWTH	New White Top-Hat
PSTNN	Partial Sum of Tensor Nuclear Norm
TLLCM	Tri-Layer Local Contrast Measure
RMSE	Root Mean Square Error
SCR	Signal-to-Clutter Ratio
SCRG	Signal-to-Clutter Ratio Gain
BSF	Background Suppression Factor
TPR	True Positive Rate
FPR	False Positive Rate
ROC	Receiver Operating Characteristic
